# Emergency Information Communication Structure by Using Multimodel Fusion and Artificial Intelligence Algorithm

**DOI:** 10.1155/2022/3029039

**Published:** 2022-10-10

**Authors:** Liping Lei

**Affiliations:** School of Political Science and Public Administration, East China University of Political Science and Law, Shanghai 201620, China

## Abstract

With the development of The Times, social events are increasing, and emergency management has gradually become the main helper to solve the crisis in the public domain. By observing the current situation of many countries and regions, we can find that various types of public crises often occur in many countries and regions in the world, which have severely affected people's daily life, lives, and property. Through long-term research and analysis, it can be known that the emergency management mechanism currently established in China has certain shortcomings. The communication problem of emergency information is likely to cause the emergency work to not proceed smoothly. In addition, problems in the communication channels of emergency information are likely to cause problems in the cooperation of various departments when people carry out emergency management work, and the efficiency of the government in dealing with problems will also be reduced in real scenarios. In order to improve the efficiency of emergency information management, this paper aims at the various problems existing and facing in the construction of emergency management system. On this basis, the integration of various relevant emergency information management plan models is analyzed and sorted out, and based on the research and integration of the development of artificial intelligence algorithms. The main research results of emergency information management at home and abroad are comprehensively studied and evaluated. Finally, a QG algorithm based on more model fusion is developed. In the process of analysis, this article uses artificial intelligence algorithms to build a prediction model of multiple modes and collects the data needed to build the model by random extraction. Through the analysis of different data sets, it is used as the basic training data for prediction. Through comprehensive analysis, the model constructed in this paper can promote the sharing of emergency information among departments to a certain extent.

## 1. Introduction

In order to improve the efficiency of problem handling as much as possible, it is necessary to enhance the effectiveness and immediacy of information communication between various government departments, whether in the beginning or the follow-up process of public crisis handling. In the process of carrying out emergency management, emergency information is the carrier of key signals transmitted by various departments, and it can also explain the connections between various subjects in public crises [1]. Improving the efficiency of communication of emergency information can promote the resolution of public crises to a certain extent. Under the current development background, various countries and regions are getting closer and closer, and many issues are related to the interests of multiple subjects [2]. Public crisis events have gradually shown new characteristics, which require all departments to do a good job of information and communication work. When dealing with emergencies, problems in the communication channels of emergency information are likely to lead to problems in the cooperation of various departments when people carry out emergency management work [3]. In reality, the efficiency of the government in handling problems will also be reduced. In actual scenarios, the transmission and sharing of information is an important way to determine whether public crisis events can be resolved quickly. Government departments must not only use professional measures to increase the speed of information transmission but also establish a crisis event alert system [4].

Due to technical difficulties, the data transmission speed and sharing function of existing information and communication systems are limited to some extent [5]. In view of the current problems, in the future of the development of information and communication system, we can rely on new technology and new theoretical knowledge to improve and optimize the development and construction of the existing information and communication management system. In order to solve the above problems and improve the efficiency of early warning and emergency information management, this paper conducts a multimodel fusion study. Combined with the development of artificial intelligence algorithms, this paper comprehensively analyzes the main achievements of emergency information management research at home and abroad and finally proposes a QG algorithm based on the multifusion model [6]. In the process of analysis, this article uses artificial intelligence algorithms to build a prediction model of multiple modes and collects the data needed to build the model by random extraction. The collected data can be used as the training data for the prediction model [7]. Ensure the scientific of the analysis results. After overall analysis, the model constructed in this article can promote the sharing of emergency information among various departments to a certain extent. Through the experimental analysis shows that through this article builds a fusion model of multiple factors, and through the use of an artificial intelligence algorithm, which is combined with the actual scene, the application of information transfer and sending and receiving efficiency and accuracy can be a certain level, to improve the communication efficiency of government departments and social crisis event comprehensive public relations capacity.

## 2. Related Work

Literature introduced the specific situation of emergency management to people, expounded on the concept and types of emergency management, and also analyzed the importance of information management to emergency management [8]. In the process of analysis, the article also introduced the grid theory, and the technology introduced by the theory can improve the effect of information integration to a certain extent and can realize information sharing and efficient dissemination between various departments. The literature also analyzes the information transmission and sharing in crisis management, the integration of various models, the development of artificial intelligence algorithms, and the main research results of emergency information management at the present stage [9]. Finally, an algorithm using multiple model fusion is proposed to improve the management efficiency of emergency information. Literature analyzes a variety of factors that affect information transmission and uses the knowledge and technology of grid theory to study the methods of information transmission and communication [10]. The article believes that the knowledge and technology of grid theory can improve the crisis. The efficiency of information management in management has confirmed the feasibility of this method based on specific analysis [11]. Literature introduced the details of information management, analyzed the development of information management and existing problems, and studied the methods of information sharing [12]. This paper conducts a series of analyses on the internal structure, operation means, and corresponding procedures of the information management system and finds that there are still a series of problems in the current system, such as uncoordinated and unbalanced communication, insufficient transparency of external information disclosure, and no connection between various communication channels [13]. Literature uses existing theories and technologies to build an information communication model. This model is mainly supported by the technology used in gridding theory [14]. In the process of research, the article also tested and analyzed the role of this model in government crisis management. Literature analyzes the practical application of gridding theory and believes that various government departments can use the advantages of gridding to realize remote office work and establish virtual information communication organizations in various regions, which can promote the improvement of information communication efficiency [15]. Help all departments and regions realize the sharing of information resources.

This article uses the knowledge of grid theory and related technologies to optimize the distribution of information communication channels and establishes a grid information-sharing system through integration. The integrated system has functions such as digital transmission, voice calls, and video chats [16]. The system can also back up various types of data at any time, realizing information sharing among different levels, different departments, and different regions. In this paper, an artificial intelligence algorithm is used to construct a fusion model of various modes in the analysis process. Under the realistic situation that the original organizational structure of the organization is controlled as much as possible without changing, the information communication channels are broadened and enriched, the level of information management is improved to a certain extent, and the quality of information management is guaranteed [17–19].

## 3. Multimodel Fusion and Artificial Intelligence Algorithm

### 3.1. Multimodel Fusion

#### 3.1.1. Sequence-to-Sequence Generative QG Model

The construction of the QG model needs to rely on the generation of the sequence. In the process of building the model, the encoder must be used to calculate the expression of the result. After the calculation is calculated, the decoder is used to sort the answer vector, so that a complete question can be generated. Let's introduce the detailed situation.

The purpose of using the encoder is to turn question-and-answer sentences with inconsistent lengths into fixed-length vectors. This type of vector is continuous, and different neural networks can be used to achieve the purpose of the encoder. The decoder will predict the problem output by the system based on the content of the sequence, which can be expressed as a mathematical formula:

(1) $$\begin{array}{c} p\left(q|a\right)=\overset{\left|q\right|}{\underset{t=1}{\prod }}p\left(q_{t}|q<t,a\right) \end{array}$$

This article uses an attention-based structure, which can calculate the probability of each word in the answer sentence. The calculation formula is as follows:

(2) $$\begin{array}{c} p\left(q_{t}|q<t,a\right)=f_{de\;c}\left(q_{t-1},s_{t},c_{t}\right) \end{array}$$

When encoding, the attention system will set a different probability or weight for each hidden state in each time step. The specific calculation formula is as follows:

(3) $$\begin{array}{c} c_{t}=\sum_{i=1}^{\left|a\right|}\alpha <i,i>h_{i} \end{array}$$

During the running of the model, the model will record the appearance of each word in different sentences, and the model will not reuse words that have already appeared. The specific calculation formula is as follows:

(4) $$\begin{array}{c} \alpha <t,i>=\frac{\exp\;\left[z\left(s_{t},h_{i},\sum_{j=1}^{N}\alpha <t-1,j>h_{j}\right)\right]}{\sum_{i^{\prime }=1}^{N}\exp\;\left[z\left(s_{t},h_{i^{\prime }},\sum_{j=1}^{N}\alpha <t-1,j>h_{j}\right)\right]} \end{array}$$

In the QG model, each component can solve the differential function, so all the parameters in the model can be learned using the backpropagation algorithm. The specific formula of the algorithm is as follows:

(5) $$\begin{array}{c} l_{qg}\left(q,a\right)=-\overset{\left|q\right|}{\underset{t=1}{\sum }}\log\;\left[p\left(y_{t}|y<t,a\right)\right]\text{.} \end{array}$$

In the process of building the model, using the grid beam search algorithm can expand the search range of the traditional beam algorithm and can calculate all the words before decoding. The algorithm can calculate the language sentences of all models. The detailed calculation formula is as follows:

(6) $$\begin{array}{c} p\left(y|X\right)={∐}_{t}p\left(y_{t}|X;\left\{y_{0}...y_{t-1}\right\}\right)\text{.} \end{array}$$

#### 3.1.2. Evaluation Index

In the process of analyzing the question types, the QGSTEC2010 assessment meeting was used in this study. The assessors can divide the test questions and sentences into five parts for scoring. The details of the scoring of the first part are shown in the following Table 1.

In the evaluation process, the question sentence needs to be converted into the corresponding query sentence, so that the system can match the corresponding data, and the output result is the answer to the question, so that it can also judge whether the type of the question is correct. The details are shown in the following Table 2.

In the process of evaluation, it is also necessary to judge whether the syntax of the question sentence is correct.

The fourth part of the assessment is the clarity of the question statement. The details are shown in the following Table 3.

**Figure 1:**
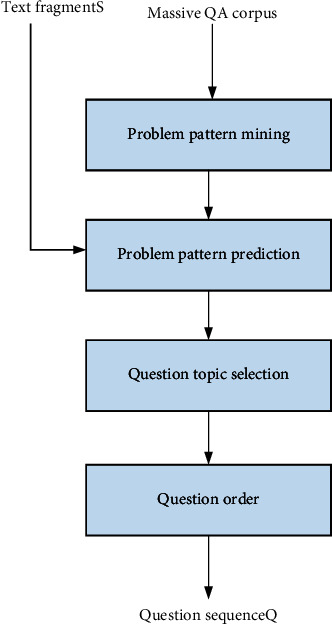
QG model framework based on problem model prediction.

In order to improve the efficiency of emergency information management, this paper analyzes various fused emergency information management scheme models on the basis of relevant problems existing in the current construction of emergency management system. Based on the research on the development of artificial intelligence algorithm, this paper refers to the main achievements of emergency information management research. Finally, a QG algorithm based on more model fusion is developed. The specific flow of the algorithm is shown in Figure 1.

In the process of designing the model, this article mainly uses the problem clustering algorithm, which can select the common problems needed to build the model from many problems. The specific calculation formula is as follows:

(7) $$\begin{array}{c} Impts\left(q_{t}^{m}\right)=\underset{q_{k}\in q_{c}}{\sum }\delta \left(q_{t}^{m},q_{k}\right)\cdot \left|q_{t}^{m}\right| \end{array}$$

The calculation formula for the elements of the model input layer is as follows:

(8) $$\begin{array}{c} Att_{i,j}=\cos\;ine\left(v_{i}^{s},v_{j}^{q_{p}}\right) \end{array}$$

In the above formula,

(9) $$\begin{array}{c} v_{k}^{s}=\max_{1<l<\left|q_{p}\right|}\left\{Att_{k,l}\right\}\\ v_{k}^{q_{p}}=\max_{1<l<\left|s\right|}\left\{Att_{l,k}\right\} \end{array}$$

Next, the attention distribution needs to be generated on each attention vector. The specific calculation formula is as follows:

(10) $$\begin{array}{c} \begin{array}{c} D_{k}^{s}=\frac{e^{v_{k}^{s}}}{\sum_{l=1}^{\left|s\right|}e^{v_{l}^{s}}}\\ D_{k}^{s_{y}}=\frac{e^{v_{k}^{q_{p}}}}{\sum_{l=1}^{\left|q_{p}\right|}e^{v_{l}^{q_{p}}}} \end{array}\text{.} \end{array}$$

After the convolution layer and pooling layer are processed in sequence, the data will be classified and packaged and transferred to the output layer. This is the expression form of the vector that requires the activation function to be calculated. The calculation formula is as follows:

(11) $$\begin{array}{c} y\left(s\right)=\tanh\;\left(W_{s}\cdot l_{p}^{s}\right)\text{.} \end{array}$$

During the operation of the convolutional layer, the composition of the data is mainly obtained by convolution calculation on the feature map and convolution kernel output by the previous layer, and the convolution kernel can be obtained through system learning. In the calculation process, different convolution kernels will produce different feature maps. The specific calculation formula is as follows:

(12) $$\begin{array}{c} L=\max\;\left\{0,M-\cos\;ine\left(y\left(s\right)\text{.}\right.\right. \end{array}$$

The encoder will read the fragments of the input layer, and the decoder will predict the output word sequence based on the read results. The specific calculation company is as follows:

(13) $$\begin{array}{c} q_{p}=\left(q_{p1},q_{p2},...,q_{p\left|q_{p}\right|}\right) \end{array}$$

In the following calculations, professional analysis tools are used to extract the candidate results of the question. If the candidate results can be extracted, the formula can be used to determine the degree of matching between the answer and the question. The specific formula is as follows:

(14) $$\begin{array}{c} s\left(q_{t},q_{p}\right)=\frac{1}{N}\cdot \underset{k}{\sum }\#\left(q_{p}^{t_{k}}\right)\cdot di\;st\left(v_{q_{i}},v_{q_{p}^{lk}}\right) \end{array}$$

If the placeholder in the formula below represents the word in the question, then this article can choose the word in the formula as the topic of the question. The specific conditions that the formula meets are as follows:

(15) $$\begin{array}{c} w_{j}=arg\max_{w_{j}\in S}\alpha_{ij}=arg\max_{w_{j}\in S}\frac{\exp\left(e_{ij}\right)}{\sum_{k=1}^{\left|S\right|}\exp\left(e_{ik}\right)}\text{.} \end{array}$$

The features required in the above formula can be combined using a linear model. The specific formula is as follows:

(16) $$\begin{array}{c} p\left(q|s\right)=\sum \lambda_{i}\cdot h_{i}\left(q,s,q_{p},q_{t}\right) \end{array}$$

### 3.2. Artificial Intelligence Algorithm

**Figure 2:**
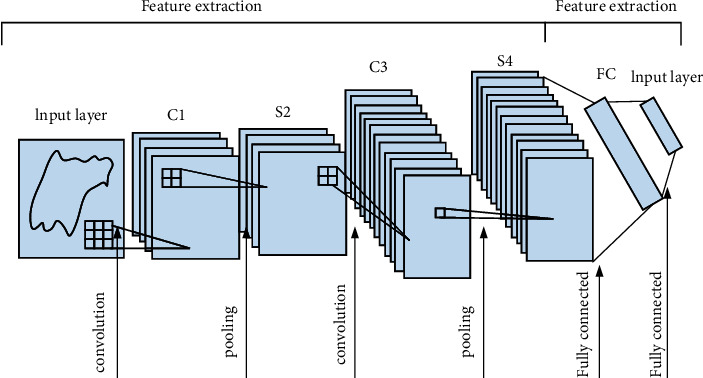
CNN network structure diagram.

The convolutional nerve is composed of a convolutional layer and a pooling layer. The two layers will alternately appear in the system as the operating cycle of the system changes. After many iterations, the system will change the pixel size of the last layer of pooling layer. It is expressed in the form of a vector, and the result is transmitted to the artificial neural network, so that the final result can be obtained. The operation process of the convolutional layer in the convolutional neural network is mainly to borrow a filter that can be used for data training to process the convolution input image and add a bias cylinder, so that the convolutional layer can be obtained. The operating process of the pooling layer is to calculate the maximum or average value of a part of pixels at intervals of a certain distance and use the calculated values to draw a feature map. Both the convolution process and the pooling process can use the activation function to activate the relevant calculation process according to the speed of the network convergence when running, so that people can extract complete image information. The specific structure of the network is shown in Figure 2.

Stacked sparse self-encoding neural network consists of two parts. The main function of the autoencoder is to extract the features contained in the image. The encoder of the neural network is composed of many sparse encoders, which can improve the accuracy of feature extraction sex. In addition, the Softmax classifier is also an important part of the neural network. The classifier can classify the extracted image features and improve the work efficiency of people analyzing image features.

#### 3.2.1. Stacked Sparse Autoencoder

**Figure 3:**
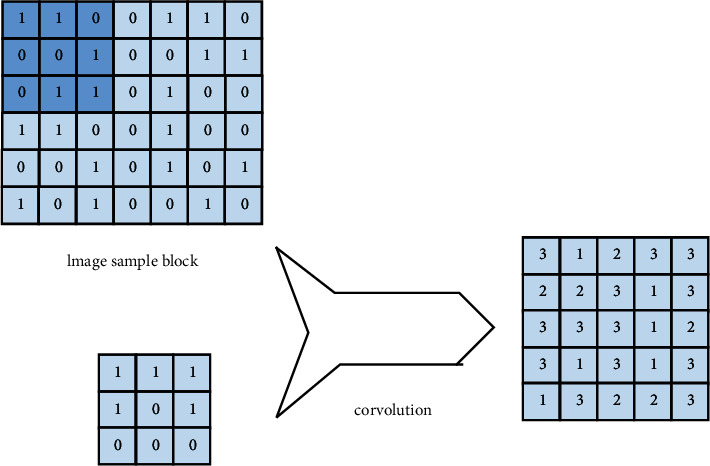
Autoencoder structure.

The autoencoder can use unsupervised learning for grid training, encode the data input to the system, restructure the data structure, and reduce the error of the restructured data, so that the characteristics of the input data in the hidden layer and the detailed structure can be obtained, as shown in Figure 3.

The hidden layer extracted by the self-encoder cannot express the data of the input layer very well. Therefore, Shausen and other related personnel have proposed a theory of sparse coding. Through the research and analysis of the brain's unsupervised learning process, they learned that the human body uses the neurons in the human body to learn external things. In fact, most of the application elements are not in the working state, some are not involved in the work, and only a small part of the neurons are activated after being stimulated, which means that the response of the neurons is incomplete. Because of this, the human brain has a better effect on learning all kinds of data information. This ability is also suitable for the autoencoders we mentioned above. With incomplete restrictions, the sparse encoder can be used for features. The sparse expression plays a good role in learning, so that the relevant information we extract is easy to distinguish. The limitation of incompleteness means that when the value of the output function of the neuron is close to 1 without limitation, the output is activated. When infinitely close to 0, the output is inhibited. In many cases, where the output is inhibited, it can be called as sparsity limitation. The specific calculation formula is as follows:

(17) $$\begin{array}{c} {\hat{\rho }}_{j}=\frac{1}{m}\overset{m}{\underset{i=1}{\sum }}\left[a_{j}\left(x^{i}\right)\right] \end{array}$$

During the operation of the network, the output of neurons will be suppressed, so the sparse activation parameters need to be used to improve the output performance of neurons, which can be expressed as

(18) $$\begin{array}{c} KL\left(\rho \|{\hat{\rho }}_{j}\right)=\rho \;\log\frac{\rho }{{\hat{\rho }}_{j}}+\left(1-\rho \right)\log\frac{1-\rho }{1-{\hat{\rho }}_{j}}\text{.} \end{array}$$

The loss function can be used to measure how the model processes the data. The specific formula is as follows:

(19) $$\begin{array}{c} J\left(W,b\right)=\frac{1}{m}\overset{m}{\underset{i=1}{\sum }}\frac{1}{2}{\left\|h_{W,b}\left(x^{i}\right)-x^{i}\right\|}^{2} \end{array}$$

After adding the penalty factor to the model, the loss function of the encoder can be expressed as

(20) $$\begin{array}{c} J_{sparse}\left(W,b\right)=J\left(W,b\right)+\beta \overset{s}{\underset{j=1}{\sum }}KL\left(\rho \|{\hat{\rho }}_{j}\right)\text{.} \end{array}$$

**Figure 4:**
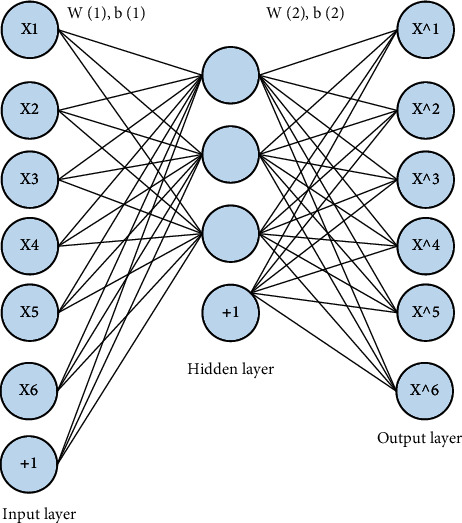
Stacked sparse autoencoder structure.

In shallow networks, sparse autoencoders can extract features that have large differences. Combining multiple sparse autoencoders to form a large encoder can improve the learning effect of deep networks. In the process of model operation, the structure of the encoder is more complicated, and the gradient disappears, which is not conducive to the training of the data, so it is necessary to adopt an unsupervised layer-by-layer greedy training method, so that the structure of the network can be optimized, thereby improving the model. The performance of the encoder is shown in Figure 4.

#### 3.2.2. Softmax Classifier

The Softmax classifier can classify the extracted image features and improve people's work efficiency in analyzing image features. When classifying, you need to use the hypothesis function to determine the characteristics of the problem. The specific formula is as follows:

(21) $$\begin{array}{c} h_{\theta }\left[x\left(i\right)\right]=\left[\begin{array}{c} p\left(y\left(i\right)\right)=\left.1\left|x\right.\left(i\right);\theta \right)\\ p\left(y\left(i\right)\right)=\left.2\left|x\right.\left(i\right);\theta \right)\\ \vdots \\ p\left(y\left(i\right)\right)=\left.k\left|x\right.\left(i\right);\theta \right) \end{array}\right] \end{array}$$

The loss function of the classifier can be determined using the maximum entropy model, which can be expressed as

(22) $$\begin{array}{c} J\left(\theta \right)=-\frac{1}{m}\left[\overset{m}{\underset{i=1}{\sum }}\overset{k}{\underset{j=1}{\sum }}1\left\{y\left(i\right)=j\;\log\frac{e^{\theta_{j}^{T}x\left(i\right)}}{\overset{k}{\underset{i=1}{\sum }}e^{\theta_{i}^{T}x\left(i\right)}}\right\}\right] \end{array}$$

In the process of data training, if the fit between the data is poor, the parameter value of the penalty function will be too large, so weights need to be used to reduce the attenuation term. At this time, the loss function can become as follows:

(23) $$\begin{array}{c} J\left(\theta \right)=-\frac{1}{m}\left[\overset{m}{\underset{i=1}{\sum }}\overset{k}{\underset{j=1}{\sum }}1\left\{y\left(i\right)=j\;\log\frac{e^{\theta_{j}^{T}x\left(i\right)}}{\overset{k}{\underset{i=1}{\sum }}e^{\theta_{i}^{T}x\left(i\right)}}\right\}\right]+\frac{\lambda }{2}\overset{k}{\underset{i=1}{\sum }}\overset{n}{\underset{j=0}{\sum }}\theta_{ij}^{2}\text{.} \end{array}$$

## 4. Research on Emergency Information Communication Structure

### 4.1. Analysis of the Status Quo of Emergency Information Communication Channels

The main objects of government emergency information communication are mostly related departments responsible for public crisis emergency management tasks. Many crisis-related data are reserved. They are important departments for the transmission and transmission of relevant emergency information. In China, the communication and exchange of this type of information generally include the following types of institutions and related personnel:

1. In China, the highest administrative leading agency for emergency management of public crises that occur suddenly is the State Council. As the highest authority, it is also the leading agency. Many affairs are discussed by its Standing Committee and presided over the work. When necessary, the relevant working groups will also be designated to guide the relevant work behaviors of other relevant agencies.
2. Main work departments. They are mainly responsible for synthesizing the relevant regulations of the government during the emergency period and then carrying out relevant coordination work. The relevant agencies responsible for these affairs are mainly the emergency offices under the State Council Office, which is an important organization that can link related affairs together.
3. Subordinate offices. Most of these institutions are responsible for dealing with relevant emergency affairs, but when dealing with these affairs, they need to carry out reasonable analysis and processing of relevant types of affairs in accordance with relevant legal regulations and their own rights. Similarly, the drafting and implementation of relevant measures are also included, and the relevant matters decided by the Party Central Committee of the State Council are implemented and carried out to the end.
4. Local institutions. This part of the agency is the secondary management agency responsible for handling the emergency management of public crises that occur suddenly. They are lower than the related agencies mentioned above and are mainly responsible for handling the relevant areas within their jurisdiction.
5. Specially dispatched expert groups and technical teams. Relevant responsible agencies generally will vigorously recruit relevant talents to form what we call the government's advisory team and will also invite and dispatch relevant experts to join relevant organizations when relevant work is needed. This is what the government faces. Put forward some valuable opinions on related issues. The government very much hopes and welcomes these professionals to actively participate in related work. In fact, when dealing with related practical issues, communication is essential, and information is needed at any time. In addition to the related institutions we have introduced above, there are many other exchanges that can be regarded as the whole of emergency information communication. These include many small temporary emergency command agencies, relevant departments such as the information early warning center, as well as some related personnel such as commanders, information officers, leaders of small teams, and related organizations, as well as some organizations with a wider range of handling such as the public and relevant media. In a state of public crisis, everyone may unwittingly become the main body of information communication. Every word spoken is a message. It may become people's concern and cause very serious consequences to other personnel. It is precisely because the scope of the subject is very wide that it should be more recognized and valued for the importance of information exchange.

**Figure 5:**
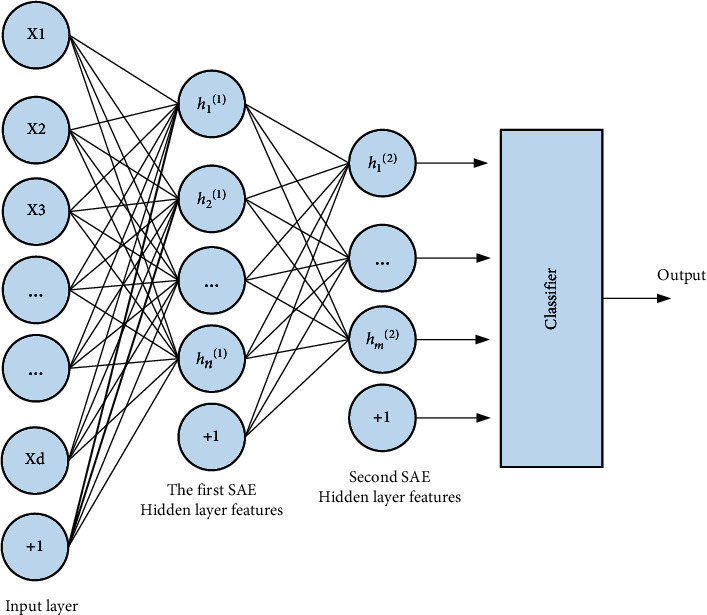
Routine general government emergency information communication process.

The determination of the government's emergency information communication process determines the government's ability and efficiency to handle public crisis events. When a public crisis event occurs, the government should reasonably arrange the order of information transmission according to the needs of the work. In the process of the Chinese government's handling of crisis events, the information communication process follows certain institutional requirements. The specific process can be divided into three steps. The details are as follows:

1. After a crisis event occurs, government departments need to report the information collected in the previous period. Once each department discovers a crisis event, it needs to report specific information to the relevant management department within four hours. In the process of handling the incident, each part also needs to report the progress of the processing in time.
2. Government departments should make preliminary responses according to the severity of crisis events. If some crisis events have a large and severe impact, each department should use their powers to initiate emergency plans and then discuss specific solutions.
3. Publicize the detailed information of the crisis event to the public, and determine the accuracy of the information before the information is released to avoid the public misunderstanding of the information. Although government departments are required to publish relevant information in a timely manner, in actual life, the government has some omissions in this aspect of work. After a crisis occurs, government departments should not only make preparations for crisis management but also verify the reliability of information through the differences and correlations of various release channels on the basis of verifying the reliable source of information, so as to ensure the authenticity of the source and content of information to the greatest extent. The specific process of government emergency information communication is shown in Figure 5.

### 4.2. Basic Model Analysis of Emergency Information Communication Channels

When constructing an information communication channel model, it is necessary to synthesize and analyze the communication channels of all information in public crisis events in various fields of society. The information communication channel is the sum of these communication channels. Government departments need to rely on information communication channels to mobilize the masses in society to cope with crisis events. This requires the government to understand the psychology and needs of the public, so as to promote the distribution and use of human resources and government funds. In the process of information transmission, multiple communication channels can improve the efficiency of information use. If information transmission and communication are not timely, it will be difficult for government departments to deal with crisis events in a short time, which is not good for the normal operation of society. From all perspectives of social development, Chinese researchers have conducted effective research on the expansion of government emergency information communication channels.

#### 4.2.1. Triangular Interactive Communication Model

This model is one of the most used models for the early handling of crisis events. This model can establish connections between government departments, the public, and the media and promote the exchange and dissemination of information among various local subjects. The social public part of the model mainly refers to the social groups that are affected when a public crisis event occurs. The government department should promptly inform this group of the progress of the crisis in the process of handling the crisis event. In addition, civil servants are a part of the groups affected by crisis events who shoulder more social responsibilities. This part of personnel should consciously fulfill their responsibility for social development.

#### 4.2.2. Regular Government Information Communication Model

The model is mainly aimed at the public and various government departments. The model designs different connections for each communication subject. Each subject can realize horizontal, vertical, and diagonal communication. Different communication methods can be connected through chains. The establishment of this model fully takes into account the government's need to deal with crisis events, and the established contacts can allow the government to communicate and exchange information with the outside world in a timely manner. In the process of government work, communication between various departments is indispensable, and government agencies and the outside public are also indispensable. Although the model has established multiple communication channels between the government and the public, the model has not tested specific performance in the actual scenarios of crisis events, which is likely to cause errors in fieldwork.

### 4.3. Model Construction of Grid Emergency Information Communication Channels

In the process of establishing grid-based information communication channels, a separate organization should be established to manage grid-based information communication channels. This organization should be composed of systematic organizational departments. Although it is a department under government management, the daily work of the organization is independent. In the process of forming the system, the supervisory center will match the supervisors according to the grid units that have been divided, so that the supervisory efficiency of the relevant areas can be guaranteed, and the supervisory axis can be formed in this way. The command center and various work departments will deal with the information of the crisis together, thus forming the axis of execution. In the grid-based communication channel, the district-level platform has two command centers, which can refine the crisis events in the city. When managing urban objects, things in the city can be materialized, and urban public facilities, road traffic, environmental protection work, emergency resource allocation, and various other types of urban things can be uniformly managed at the same time.

In the work process of the government, each divided area should carry out corresponding activities according to the requirements of the work, and the supervision and implementation of each part should cooperate with each other. When the command center and the control center release and inspect the work of each department, they should let the monitoring axis cooperate with the specific work of the axis. The two centers should always issue reasonable tasks to each area and supervise each area to perform tasks. Through specific practice, we can know that all parts of the system are connected to a certain degree, which can ensure that there will be no omissions in the process of information communication, and each part of the system can check the content of the information. Once a crisis event occurs, each part information will be released to the center of the system, so that the efficiency of government departments in handling crisis events can be improved.

## 5. Conclusion

In the development of recent years, many countries and regions in the world often have various types of public crises, which have severely affected people's daily life, lives, and property. Through long-term research and analysis, it can be known that the emergency management mechanism currently established in China has certain shortcomings. The communication problem of emergency information is likely to cause the emergency work to not proceed smoothly. In addition, problems in the communication channels of emergency information are likely to cause problems in the cooperation of various departments when people carry out emergency management work, and the efficiency of the government in dealing with problems will also be reduced in real scenarios. In the process of analyzing related issues, this article elaborates on the concept of information communication channels, the concept of emergency management, the principles of information communication, and the characteristics of information sharing. The article believes that the knowledge and technology can provide a good help for the communication and sharing of information. When dealing with emergencies, problems in the communication channels of emergency information are likely to lead to problems in the cooperation of various departments when people carry out emergency management work. In reality, the efficiency of the government in handling problems will also be reduced. In actual scenarios, the transmission and sharing of information is an important way to determine whether public crisis events can be resolved quickly. Government departments must not only use professional measures to increase the speed of information transmission but also establish a crisis event alert system based on the characteristics of the information. This ensures that the crisis management department can grasp the detailed incident information in a timely manner, and ensure that the public can understand the specific situation of incident handling. This article also analyzes the problems that Chinese government departments have in the process of handling public crises and provides a reasonable plan for information communication and sharing between various departments and subjects. In the future development process, an emergency information management system based on multimodel fusion and artificial intelligence algorithms will provide more convenience to the work of government departments.

## Figures and Tables

**Table 1:** QGSTEC2010 one of the evaluation standards: whether the question is related.

1	The generation question is completely related
2	The generation problem is basically related
3	The generation problem is basically irrelevant
4	The generation problem is completely irrelevant

**Table 2:** One of QGSTEC2010 evaluation standards: is the question type correct?

Sort	Description
1	The generated problem is of a given type
2	The generation problem is not a given type

**Table 3:** One of QGSTEC2010 evaluation standards: is the problem clear?

Sort	Description	For example
1	More information is needed to clarify the meaning of the problem	Who was nominated in 1997?
2	When there is no context, the answer is ambiguous	Who was nominated?

## Data Availability

The data used to support the findings of this study are available from the corresponding author upon request.
